# Time-dependent mechanical behaviour of the aortic chronic dissection flap

**DOI:** 10.1093/icvts/ivac029

**Published:** 2022-02-10

**Authors:** Phakakorn Panpho, Ying Yang, Hannah A Davies, Omar Nawaytou, Amer Harky, Francesco Torella, Mark Field, Jillian Madine, Riaz Akhtar

**Affiliations:** 1 Faculty of Science and Technology, Pibulsongkram Rajabhat University, Phitsanulok, Thailand; 2 Department of Mechanical, Materials and Aerospace Engineering, School of Engineering, University of Liverpool, Liverpool, UK; 3 School of Pharmacy and Bioengineering, Keele University, Stoke-on-Trent, UK; 4 Department of Cardiovascular and Metabolic Medicine, Institute of Life Course and Medical Sciences, Faculty of Health and Life Sciences, University of Liverpool, Liverpool, UK; 5 Liverpool Centre for Cardiovascular Science, Liverpool, UK; 6 Department of Cardiac Surgery, Liverpool Heart and Chest Hospital, Liverpool, UK; 7 Liverpool Vascular & Endovascular Service, Liverpool, UK; 8 Department of Biochemistry and Systems Biology, Institute of Systems, Molecular and Integrative Biology, Faculty of Health and Life Sciences, University of Liverpool, Liverpool, UK

**Keywords:** Chronic aortic dissection, Dissection flap, Biomechanics, Biochemistry, Time-dependent deformation, False lumen

## Abstract

**OBJECTIVES:**

The transition of aortic dissection from acute to chronic is poorly understood. We examined time-dependent mechanical behaviour and biochemical properties of chronic dissection tissues.

**METHODS:**

Aorta samples were obtained from 14 patients with mixed aetiology who were undergoing elective surgery for chronic dissected aneurysms, ranging from 3 months to 15 years post-dissection. The tissue elastic modulus and tissue deformation following application of loading for 5 h were measured for the false lumen (FL), true lumen (TL) and flap (FP) tissues with a custom-indentation technique. Collagen, elastin and glycosaminoglycan levels were determined with established biochemical assays. Elastin fragmentation was graded from histological sections. The number of tissues characterized was as follows: FP (*n* = 10), TL (*n* = 5 for biomechanical testing, *n* = 8 for biochemical analysis, *n* = 8 for histological assessment) and FL (*n* = 4).

**RESULTS:**

Tissues stiffness was highest in FP [59.8 (14.8) kPa] as compared with TL [50.7 (6.2) kPa] and FL [40.5 (4.7) kPa] (*P* = 0.023 and *P* = 0.006, respectively). FP [0.5 (0.08) mm] also exhibited reduced deformation relative to TL [0.7 (0.02) mm] and FL [0.9 (0.08) mm] (*P* = 0.003 and *P* = 0.006, respectively), lowest collagen concentration [FP: 40.1 (19.6) µg/mg, TL: 59.9 (19.5) µg/mg, *P* = 0.008; FL: 79.1 (32.0) µg/mg, *P* = 0.006] and the lowest collagen: elastin ratio [0.4 (0.1)] relative to the other tissues [TL; 0.6 (0.3), *P* = 0.006, FL; 1.5 (0.4); *P* = 0.003]. Significant elastin loss was evident in the FL-stained tissue sections whereas highly aligned, long fibres were visible in the FP and TL. A linear relationship was found between the stiffness, deformation and the time from the dissection event to surgical intervention for the FP. All data are presented as median (interquartile range).

**CONCLUSIONS:**

FP exhibited reduced time-dependent deformation and distinct biochemical properties relative to TL and FL irrespective of connective tissue disorder or the anatomical region of the dissection.

**Figure:**
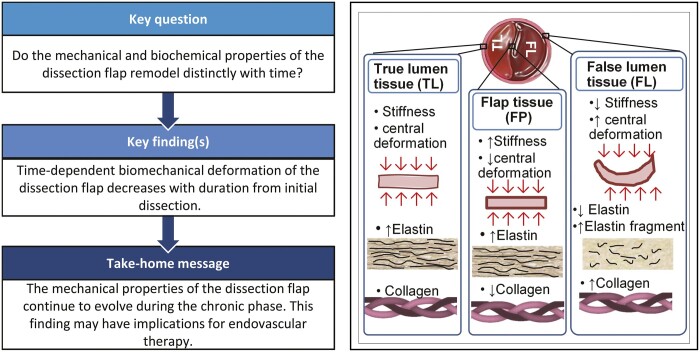

## INTRODUCTION

Aortic dissection (AD) is characterized by the pathological formation of a new vascular lumen within the tunica media, and can be acute or chronic. The transition between the 2 stages, or sub-acute phase, is ill-defined, with no agreed duration. The European Society of Cardiology Task Force for the Diagnosis and Treatment of Aortic Disease defines AD as sub-acute between 15 and 90 days after onset [1], whereas some investigators have deemed AD sub-acute up to 52 weeks after onset [2]. Regardless of its duration, during the sub-acute phase of AD, the dissection flap (FP) and the dissected aorta undergo progressive structural changes, which cause, in many cases, progressive dilatation of the false lumen (FL), leading to death or intervention in more than half of patients managed conservatively at presentation [3]. Stanford type B ADs are often treated by endovascular means, particularly in the acute (when complicated) and sub-acute phases, when stenting of the true lumen (TL) is performed to induce FL collapse and remodelling of the whole aorta around the endoprosthesis [4]. This treatment is effective in inducing remodelling and reducing the risk of late complications [5]. Although remodelling can also occur in the chronic dissection phase [6] once chronicity is established; however, stenting is unlikely to induce complete FL collapse, as the initially mobile FP loses plasticity and stiffens. Any treatment of AD in the chronic phase, whether endovascular or surgical, is thus simply aimed at preventing or managing aortic rupture, by excluding aneurysmal segments from the main circulation. Understanding the biomechanics of dissected aortic tissues is critical to therapeutic decisions: it is theoretically possible that the window of opportunity for inducing aortic remodelling by endovascular stenting of the TL (the sub-acute phase) varies from case to case if structural changes in AD tissues do not occur at the same speed in every patient, due to differences in phenotype and/or the pathological process. Such changes may even be patient-specific. In fact, a recent study has shown the potential of *in vivo* assessment of FP mobility and dynamics prior to thoracic endovascular aortic repair with transoesophageal echocardiography analysis [7]. FP mobility was found to correlate to aortic remodelling after thoracic endovascular aortic repair and the authors highlight the benefit of intraoperative assessment and inclusion of dissection FP motility parameters in surgical decision-making. Given these findings, one would expect heterogeneous biomechanical, biochemical and histological features in chronic AD tissues. Here, we aimed to determine the elastic and time-dependent biomechanical properties of chronic dissection tissues, including the FP and the walls of the aortic lumina, and to relate these to tissue biochemistry and histology, as well as to the interval between onset of AD and surgery.

## METHODS

### Ethical statement

The study protocol was approved by the Liverpool Bio-Innovation Hub (LBIH) biobank (project approval references 15-06 and 19-09). The LBIH Biobank confers ethical approval for the use of samples as a Research Tissue Bank (REC reference 14/NW/1212, NRES Committee North West–Haydock).

### Tissue and patient characteristics

A segment of fresh aortic wall was excised from resection surgery obtained from 14 consenting patients undergoing elective surgical repair for chronic dissecting aneurysm at Liverpool Heart Chest Hospital (LHCH; Table 1). The Interval of index event to operation (IIEO) defined as the duration from the dissection event to surgical intervention was obtained from patient records.

**Table 1:** Summary of patient clinical characteristics collated from electronic patient records along with the tissue types per patient, and the experimental techniques conducted

Patient ID	Age, Year	Gender	Syndromic (Marfan)	Hypertension	Hypercholesterolemia	Family history of aneurysm	Tissue type	Ball indentation	Collagen Level	GAG Level	Elastin Level	Elastin fragmentation
894-15	65	M	N	Y	Y	N	TL	X	✓	✓	✓	✓
05-00001-16	45	M	Y	N	N	N	FP	✓	✓	✓	✓	✓
05-00020-16	49	M	N	N	N	N	FP	✓	✓	✓	✓	✓
							TL	✓	✓	✓	✓	✓
05-00023-16	37	M	Y	Y	N	Y	TL	X	✓	✓	✓	✓
05-00027-16	66	M	N	Y	Y	N	TL	✓	✓	✓	✓	✓
05-00045-16	68	M	N	Y	Y	N	FP	✓	✓	✓	✓	✓
05-00064-16	54	M	Y	N	N	N	FP	✓	✓	✓	✓	✓
05-00070-16	62	M	N	N	N	N	FP	✓	✓	✓	✓	✓
							TL	X	✓	✓	✓	✓
05-00020-17	77	M	N	Y	Y	N	FP	✓	✓	✓	✓	✓
05-00070-17	50	M	Y	N	N	N	FL	✓	✓	✓	✓	✓
05-00003-18	40	M	N	N	Y	N	FP	✓	✓	✓	✓	✓
							TL	✓	✓	✓	✓	X
							FL	✓	✓	✓	✓	✓
05-00040-18	60	M	N	Y	Y	N	FP	✓	✓	✓	✓	✓
05-00064-18	39	M	Y	Y	N	Y	FP	✓	✓	✓	✓	✓
							TL	✓	✓	✓	✓	✓
							FL	✓	✓	✓	✓	✓
05-00017-19	67	M	N	Y	Y	N	FP	✓	✓	✓	✓	✓
							TL	✓	✓	✓	✓	✓
							FL	✓	✓	✓	✓	✓

In total, there were 14 patients and 22 samples split into FP, TL and FL. For 3 patients, all 3 tissues (FP, TL and FL) were obtained at the time of surgery. Only tissue from 12 patients was available for the biomechanical testing.

FL: false lumen; FP: flap; GAG: glycosaminoglycan; TL: true lumen.

**Table 2:** Clinical characteristics for the patients, grouped by tissue type

	FP	TL	FL
Biochemistry samples, ***n***	10	8	4
Biomechanics samples, ***n***	10	5	4
Age	57(22)	55.5(26)	45(19)
		49(26)	
Gender			
Male, ***n***	10 (100%)	8 (100%)	4 (100%)
		5 (100%)	
Female, ***n***	0	0	0
Location			
Ascending	1 (10%)	1 (12.5%)	2(50%)
		1(20%)	
Descending	9 (90%)	7 (87.5%)	2 (50%)
		4(80%)	
Preoperative aortic diameter (cm)	5.35(0.8)	5.15(1.9)	5.25(1.8)
		5.2(2.0)	
Aetiology			
Syndromic (Marfan)	3 (30%)	2 (25%)	2 (50%)
		1(20%)	
Non-syndromic (unknown)	7 (70%)	6 (75%)	2 (50%)
		4(80%)	
Family history of aneurysm			
Yes, ***n***	1 (10%)	2 (25%)	1 (25%)
		1(20%)	
No, ***n***	9 (90%)	6(75%)	3 (75%)
		4(80%)	
Hypertension			
Yes, ***n***	5 (50%)	5 (62.5%)	2 (50%)
		3(60%)	
No, ***n***	5 (50%)	3 (37.5%)	2 (50%)
		2(40%)	
Hypercholesterolemia			
Yes, ***n***	5 (50%)	4 (50%)	2 (50%)
		3 (60%)	
No, ***n***	5 (50%)	4 (50%)	2 (50%)
		2 (40%)	

Data are displayed as median (IQR) values and ***n*** represents number of patients. The second row in the TL column refers to the values for the samples available for biomechanical testing.

FL: false lumen; FP: flap; TL: true lumen.

The tissues were grouped into 3 main types: FP (*n* = 10), TL (*n* = 5 for biomechanical testing, *n* = 8 for biochemical analysis, *n* = 8 for histological assessment) and FL (*n* = 4). Table 2 presents the patient clinical characteristics grouped by tissue type. For biomechanical testing, a 1.5 × 1.5 cm sample of tissue was cut and any excess connective and adipose tissue was removed. The samples were embedded in optimal cutting temperature embedding medium (Cellpath™ OCT Embedding Matrix) and immediately snap-frozen using dry ice and super-cooled isopentane for cryosectioning. Cryosections with a thickness of 200 µm were cut using a Thermo Scientific™ Microm HM525 NX Cryostat (Fisher Scientific UK Ltd, Loughborough, UK) and adhered onto glass slides. The entire thickness of the sample was sectioned and only the central part of the tissue was utilized. All cryosections were kept at −80°C until tested. Prior to testing, the sections were thawed at room temperature for 5 min and then washed with distilled water to remove any excess optimal cutting temperature.

### Biomechanical testing with ball indentation

**Figure 1:**
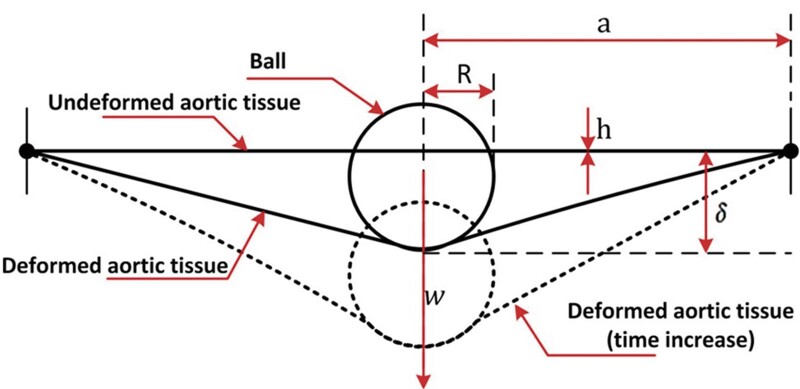
Schematic representation of indentation of the aortic tissue by the weight of spherical ball. The figure is adapted from Ref. [8].

The time-dependent mechanical behaviour and elastic modulus (E) of the aortic tissues were investigated using a non-destructive ball indentation method based on a pre-established device by other researchers [8, 9]. The setup is described in detail in the Supplementary Material, Fig. S1. The E was determined with Equation (1) using a method previously applied to biological tissue [9] and hydrogels [8].

(1) $$E=\frac{6w}{\text{hR}}*\frac{1}{0.075{\left(\frac{\delta }{R}\right)}^{2}+0.78(\frac{\delta }{R})}\;,$$

where *w* is the weight of the spherical ball, *h* is the thickness of the aortic tissues, *R* is the radius of spherical ball and δ represents the initial displacement caused by the weight of the spherical ball (Fig. 1).

Equation (1) is valid (applicable) for a ball and a membrane-like tissue with the dimensional characteristics of a/R = 5 and δ/R = 1.7, where a is the radius of the tissue. It is also assumed that the ratio of thickness to the radius is low and the deformation is large, hence stretching of the tissue dominates over bending [8].

**Figure 2:**
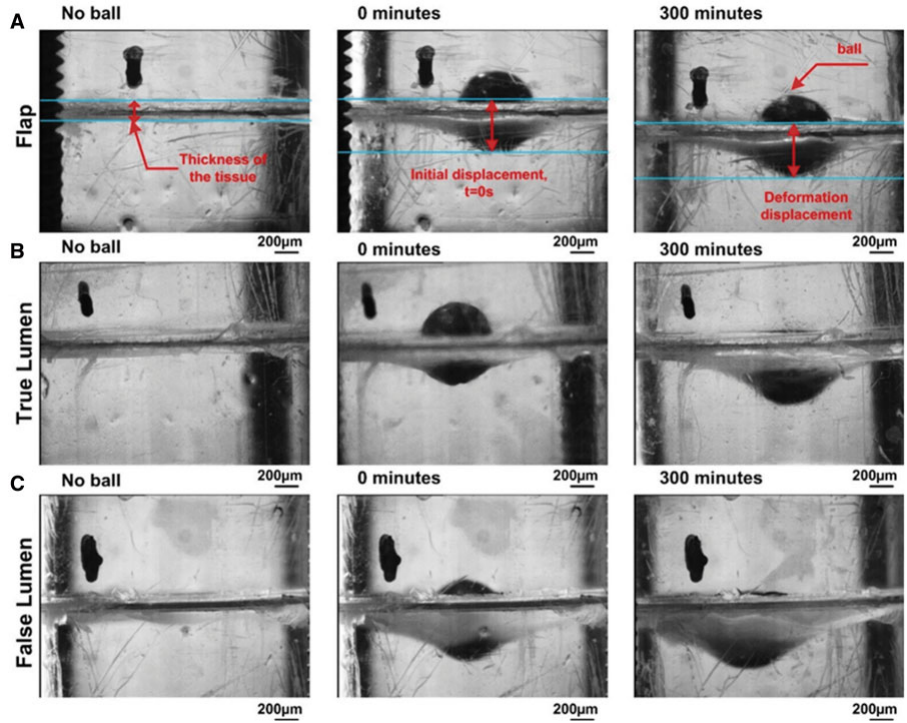
Images showing tissue deformation with no ball, at 0 and 300 min: (**A**) flap, (**B**) true lumen and (**C**) false lumen. Scale bar represents 200 µm.

A side view of the deformed chronic AD tissues was captured by a long focal microscope as shown in Fig. 2. The initial displacement at 0 min was used to obtain E. Time-dependent deformation of the tissue, referred to as central deformation, was recorded over 300 min. Central deformation against time gives an indication of creep which is tendency of a material to deform under the influence of a constant mechanical stress. In the case of the dissection tissues, it enables quantification of tissue ‘mobility’ *in vitro*.

### Biochemical analysis

The tissues samples (FP, TL and FL) were digested by either papain solution to extract for glycosaminoglycans (GAGs) and collagen, or oxalic acid to extract for elastin. The papain-digested specimens were assayed as triplicates and the oxalic acid-digested specimens were assayed as duplicates. Collagen levels were determined by the concentration of hydroxyproline within the samples and compared with L-hydroxyproline [10, 11]. The Fastin Elastin Kit (Biocolor, Carrickfergus, UK) protocol was followed to determine α-elastin levels within the tissue samples. Dimethyl methylene blue dye was used to detect sulphated GAG levels within the samples and compared against the standard; Chondroitin Sulphate C [12]. Further details including reagents used and their concentrations can be found in the Supplementary Material.

### Histological analysis

For histological staining, sections were deparaffinized, dehydrated and subjected to Verhoeff–Van Gieson staining using standard kits according to manufacturer’s protocols. Images were generated using an Axio slide scanner (Carl Zeiss Microscopy, White Plains, NY, USA) at 20x resolution. Four random rectangle images at the medial layer were taken per tissue to grade elastin fragmentation, defined as focal fragmentation of elastin. The criteria for the histologic grading were modified from Schlatmann and Becker [13] (see Supplementary Material).

### Data processing and statistical analyses

Values in the text are presented as median (interquartile range). Statistical analysis was performed using OriginPro version 9 (OriginLab, Northampton, MA, USA). The biomechanical properties (E and central deformation) and biochemical properties (collagen, GAG and elastin level) for each group: FP, TL and FL are presented using box plots with 5th and 95th percentile as whiskers. For all comparisons between each group, the Mann–Whitney *U*-test was performed. Bivariate correlation with Spearman product-moment correlation coefficients was calculated. This was performed to define the relationship between E, biochemical properties (collagen, GAG elastin and collagen/elastin ratio level) and histological data with IIEO. *P* < 0.05 was considered as statistically significant.

## RESULTS

### Patients and demographics

All 14 patients were male, with ages ranging from 37 to 77 years (median; 52 years). Five had Marfan’s syndrome. The remaining 9 were classified as non-syndromic. Tissue was harvested from the ascending (*n* = 2) or descending (*n* = 12) aorta. Two of the 14 patients had a family history of aneurysm (14.3%); 8 of 14 patients had hypertension (57.1%) and 7 of 14 patients had hypercholesterolaemia (50%).

### Biomechanical and biochemical findings

**Figure 3:**
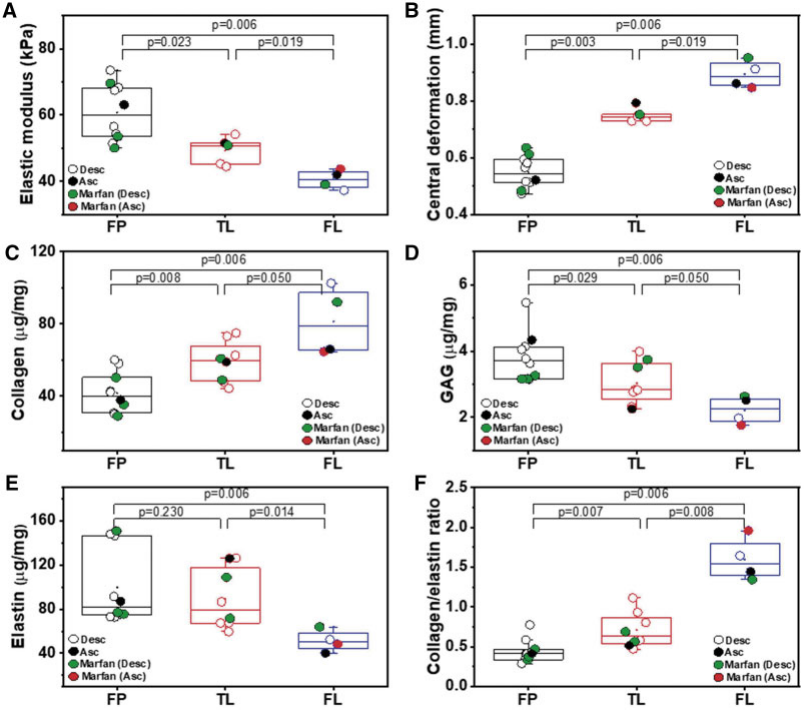
Box plots of biomechanical and biochemical data for the FP, TL and FL. All data are represented as box plots representing the 25th and 75th percentiles of data. Whiskers represent the 5th and 95th percentiles of data and the middle horizontal lines represent median values. Each point within the plot represents an individual tissue for the variable specified. Variables acquired from the specimens were: (**A**) E, (**B**) central deformation, (**C**) collagen levels, (**D**) GAG level, (**E**) elastin levels and (**F**) collagen/elastin ratio. Mann–Whitney analyses were performed to determine statistical difference between groups. Desc and Asc are descending and ascending regions, respectively. Red points represent tissue from a Marfan’s syndrome patient where the sample was harvested from the descending region. Green points also represent a Marfan’s patient but where the tissue is harvested from the ascending region. FL: false lumen; FP: flap; GAG: glycosaminoglycan; TL: true lumen.

E for FP, TL and FL is presented in Fig. 3A. Overall, significantly greater E (tissue stiffness) was observed in FP [59.8 (14.8) kPa] as compared with TL [50.7 (6.2) kPa] and FL [40.5 (4.7) kPa] (*P* = 0.023 and *P* = 0.006, respectively). E was significantly lower in FL relative to TL (*P* = 0.019). Central displacement was also significantly different when comparing FP [0.5 (0.08) mm] and TL [0.7 (0.02) mm, *P* = 0.003], FP and FL [0.9 (0.08) mm, *P* = 0.006], as well as TL and FL (*P* = 0.019). It was noticed that the central displacement for FP was the lowest relative to TL and FL (Fig. 3B).

Collagen, elastin and GAG levels were quantified and presented in Fig. 3C–E. The collagen level was significantly higher in FL [79.1 (32.0) µg/mg] as compared with TL [59.9 (19.5) µg/mg, *P* = 0.032] and FP [40.1 (19.6) µg/mg, *P* = 0.003] (Fig. 3C) whereas GAG level for FL [2.3 (0.7) µg/mg] was significantly lower relative to TL [2.8 (1.1) µg/mg, *P* = 0.016] and FP [3.7 (1.0) µg/mg, *P* = 0.003] (Fig. 3D). Elastin levels were marginally greater in TL [78.9 (49.9) µg/mg] relative to FP [81.7 (71.4) µg/mg, *P* = 0.270] but not significantly. The lowest elastin level and narrow distribution were noticed in FL [50.3 (13.9) µg/mg] as compared with FP and TL (Fig. 3E). Collagen/elastin ratio was lowest in FP [0.4 (0.1)] relative to TL [0.6 (0.3), *P* = 0.006] and FL [1.5 (0.4), *P* = 0.003] (Fig. 3F).

### Histological analysis

#### Elastin fragmentation

**Figure 4:**
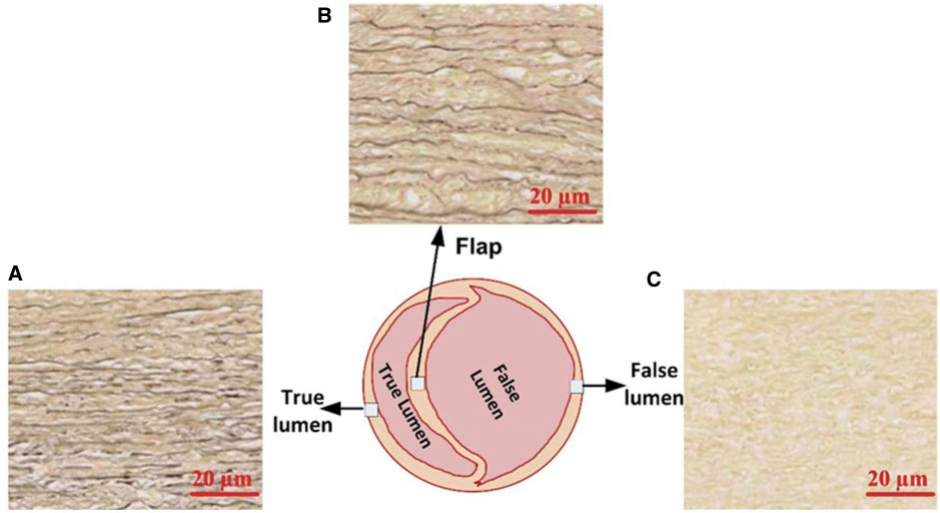
Example elastin fragmentation observed using Verhoeff–Van Gieson stained sections from chronic aortic dissection tissues: (**A**), (**B**) and (**C**) demonstrate true lumen, flap and false lumen, respectively. Scale bar is 20 µm.

Representative Verhoeff–Van Gieson stained sections for the TL, FP and FL shown in Fig. 4. The TL and FP tissues had highly compact, long, aligned elastic fibres as compared with FL tissues.

**Figure 5:**
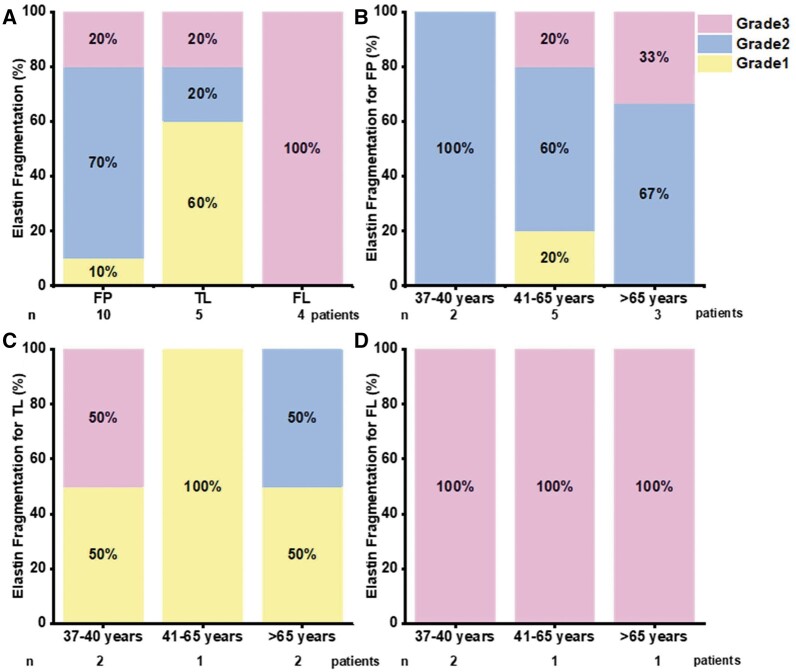
Overall percentages for each tissue (**A**) and percentage of elastin fragmentation graded by age for (**B**) FP, (**C**) TL and (**D**) FL. FL: false lumen; FP: flap; TL: true lumen.

Sixty per cent of the TL sections were classified as Grade I, whilst for the FP the sections were classified as Grade II for 70% of the images. All of the FL sections were classified as Grade III (Fig. 5A). Given that elastin fragmentation occurs in the aortic wall with increasing age [13] and this information is important, the graded images were also classified by age as shown in Fig. 5B–D.

### Correlations of biomechanical data with interval of index event to operation

**Figure 6:**
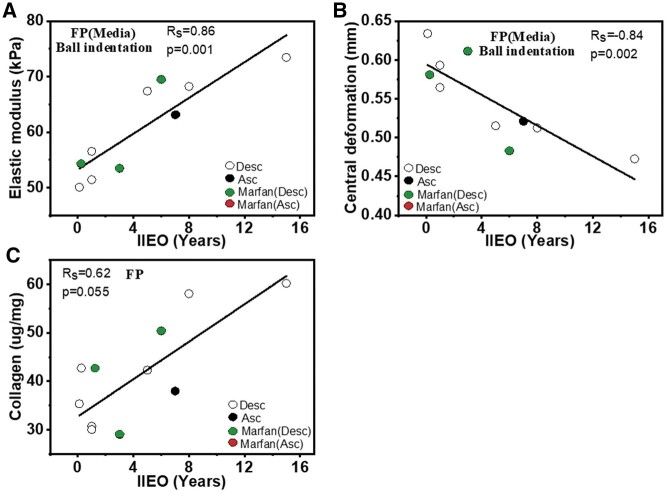
Relationships between biomechanical and biochemical findings with IIEO for FP (*n* = 10) showing (**A**) E, (**B**) central deformation, (**C**) collagen concentration. Additional correlations are shown in Supplementary Material, Figs. S2 and S3. FP: flap; IIEO: interval of index event to operation.

The mean IIEO was 4 years, with a minimum of 3 months and a maximum of 15 years. Correlations between biomechanical properties with IIEO are shown for all groups in Supplementary Material, Fig. S2. The most important trends are shown in Fig. 6. There was a significant positive correlation between E with IIEO for FP (Rs = 0.86 and *P* = 0.001; Fig. 6A), and a strong negative relationship between central deformation for FP tissues and IIEO (Rs = −0.84 and *P* = 0.002; Fig. 6B). Collagen, GAG, elastin and collagen/elastin ratio were also compared to IIEO (Supplementary Material, Fig. S3). A positive correlation was observed between collagen concentration with IIEO (Rs = 0.62 and *P* = 0.055; Fig. 6C); however, this was not significant (*P* > 0.05).

## DISCUSSION

Degeneration of the dissected aorta is characterized by progressive FL dilatation. Our study demonstrates the biomechanical changes in the properties of the aortic wall underpinning this change, which is related to greater compliance of the FL. In turn, biomechanical degeneration appears to be caused by a loss of elastin and elastin fragmentation, which is most apparent in the FL. Our data also suggest that aortic degeneration in AD occurs independently of the presence of an underlying connective tissue disorder. Finally, our analysis elucidates the mechanism leading to progressive stiffness of the FP over time. In fact, stiffness (high E) was highest and deformation lowest in FP relative to TL and FL, and central deformation of FP was significantly lower than in TL and FL.

The biomechanical characteristics of the FP were related to densely packed elastin fibres with little fragmentation. Interestingly, whilst FP increases in stiffness in time, the biomechanics of the TL and FL do not appear to depend on IIEO.

In patients with IIEO >5 years, central deformation was minimal but still detectable, with no deformation observed for the patient with IIEO of 15 years. This finding suggests that some aortic remodelling post-AD may be expected to occur even after several months or a few years post-onset, hence that the transition between the sub-acute and chronic phase is a gradual phenomenon, which is only fully complete after several years of onset. To date, there are limited studies in the literature on the behaviour of human dissected tissues. A recent study demonstrated that chronic FPs exhibit increased stiffness and decrease compliance, supporting our findings [14]. These findings are also in line with clinical imaging observations which suggest that the FP becomes straighter, with increased stiffness (immobility) as acute AD transitions over time to chronic AD (1 year) [15].

The stiffness of the FP and FL compliance could be explained by understanding the elastin distribution and architecture seen via histological images. The elastic fibres in TL and FP tissues were highly compact close to the intima layer, with long and aligned elastin fibres observed, similar to findings by Moriwaki and co-workers [16]. They reported that the E of collagen-rich regions was substantially lower than that of the elastin-rich regions, despite collagen fibrils having a significantly higher E relative to elastin [16]. This finding was also reported by Matsumoto *et al.* [17], who found that the high E region of the porcine aortic wall is mainly composed of elastin and that the low E region mostly comprises smooth muscle cells. We found that the FL is more compliant and explained by the loss and fragmentation of elastic fibres. We also found that collagen was highest in the FL relative to the FP and TL, although the compliance was highest in the FL. Collagen is associated with imparting tensile strength to tissue and associated with tissue stiffness. However, the high stiffness of the FP with the lower concentrations can be understood by the likelihood of increased crosslinking and fibrosis, which is not measured with the hydroxyproline assay used. In addition, the type of collagen present will impact on mechanical behaviour. Type III collagen imparts extensibility whilst Type I imparts stiffness [18, 19]. The type of collagen present in the dissected tissues would warrant further investigation. Biomechanical changes in this study appear to be driven by the role of elastic fibres alignment, architecture and degradation in aortic wall. These findings are, to the best of our knowledge, the first report on the local E of chronic AD wall tissues.

Our findings offer a biomechanical and pathological explanation of the therapeutic effects of endovascular therapy of AD. In the acute and sub-acute phase, stents are used to cover intimal tears and to expand the TL, thus inducing secondary collapse of the FL. This can only be achieved if there is limited stiffness of the FP [7]. As the collagen composition of the FP changes over time, stiffness increases and compliance decreases, progressively requiring greater radial forces to achieve full TL re-expansion. The sub-acute phase could thus be defined, in biomechanical terms, as the period during which full TL expansion is potentially achievable by endovascular means. Notably, this phase would depend on the radial force of available stent-grafts as well as on the biomechanical characteristics of the FP.

Interestingly, the biomechanical data are independent of tissue aetiology (Marfan syndrome or non-syndromic) or location (ascending or descending). This finding suggests that endovascular therapy should have the same effects on TL re-expansion in syndromic and non-syndromic patients. Nevertheless, the choice of therapy should not be simply dictated by the likelihood of response in the dissected segment but also by the likelihood of late dissection and expansion of the adjacent normal aorta (the landing zones), which is significantly greater in patients with connective tissue disorders. In this group of patients, surgical therapy may prevent late re-perfusion of the FL in segments treated endovascularly, when proximal dissection occurs, as occasionally observed in our practice and by other authors [20].

### Limitations

Our limited sample size with a male only cohort allows limited extrapolation to the general AD population. Similar studies on larger numbers would be needed to confirm our findings and to define the evolution of biomechanical properties of dissected tissues over time. Such studies may allow the re-definition of the sub-acute phase of AD on the basis of the likelihood of response to endovascular therapy, if coupled with bench testing on the effects of the forces generated by commercially available stent grafts on tissues with various stiffness/compliance. Further, it would be interesting to correlate histological and biomechanical features of the FP to its mobility through the cardiac cycle as this cannot be visualized with dynamic diagnostic modalities such as magnetic resonance and ultrasound.

Inflammatory reactions are likely involved in the development of AD and exhibit different time courses of their changes in acute-phase reactions and between acute and chronic AD, but this is beyond the scope of this paper. Further, in order to gain insight into the FP behaviour at the hinge points, the biomechanical properties of FP at the hinge points should be investigated. Finally, given assumptions with the use of Equation (1), E values should not be considered absolute but do show comparative trends.

## CONCLUSIONS

Our study probed biomechanical and biochemical changes in chronic AD tissues (FP, TL and FL) and their relationship with IIEO. We show a loss of tissue compliance in FP and TL relative to FL, with FP being the stiffest. Loss of tissue compliance in FP and TL was related to compact, long and aligned elastic fibres, whereas the FL was highly compliant with high elastin fragmentation. Such changes were unrelated to the presence of underlying connective tissue disorders. This study contributes to understanding of how the structural properties of dissection tissues within the aortic wall alter with time and may explain why and how endovascular therapy works in various phases of AD.

## SUPPLEMENTARY MATERIAL

Supplementary material is available at *ICVTS* online.

## Supplementary Material

ivac029_Supplementary_Data

## Notes

Presented at the 35th Annual Meeting of the European Association for Cardio-Thoracic Surgery, Barcelona, Spain, 13–16 October 2021.

## ABBREVIATIONS

AD: Aortic dissection
E: Elastic modulus
FL: False lumen
FP: Flap
GAG: Glycosaminoglycan
IIEO: Interval of index event to operation
TL: True lumen aortic wall
