# Supplementary material for: Time-dependent mechanical behaviour of the aortic chronic dissection flap
Source: Interact Cardiovasc Thorac Surg. 2022 Feb 10;34(5):892–901. doi: 10.1093/icvts/ivac029 (PMC9070530; doi:10.1093/icvts/ivac029)
Supplement: ivac029_Supplementary_Data [file ivac029_supplementary_data.docx]

**Supplementary Information**

The set-up for a non-destructive ball indentation method is composed of two main parts: 1) a sample holder with a specific design includes two transparent plastic circle rings clamped together with stainless steel plates (Figure S1A and B) and 2) an image acquisition and processing system (Figure S1C). The aortic section was clamped between two transparent rings and then placed on the stainless-steel plate above the transparent ring and screwed to it. Deformation of the tissues was achieved using a constant stainless-steel ball of weight 0.079 g and diameter 2.4 mm which was placed on the central portion of the tissues. The whole assembly of the sample holder were placed in a moist square petri dish and incubated at 37^o^C and 5% CO_2_. The side view of the tissue deformation was imaged and recorded with the acquisition system which was composed of a long focal distance microscope (Edmund Optics, Barrington, USA) connected to a CCD video camera (Sony CCD XC-ST50CE Video Camera Module, Japan). The software module employed in this work for displaying and recording the images was developed in LabView (National Instruments Corporation (U.K.) Ltd., Berkshire). Creep deformation of the tissue was recorded over 300 minutes.


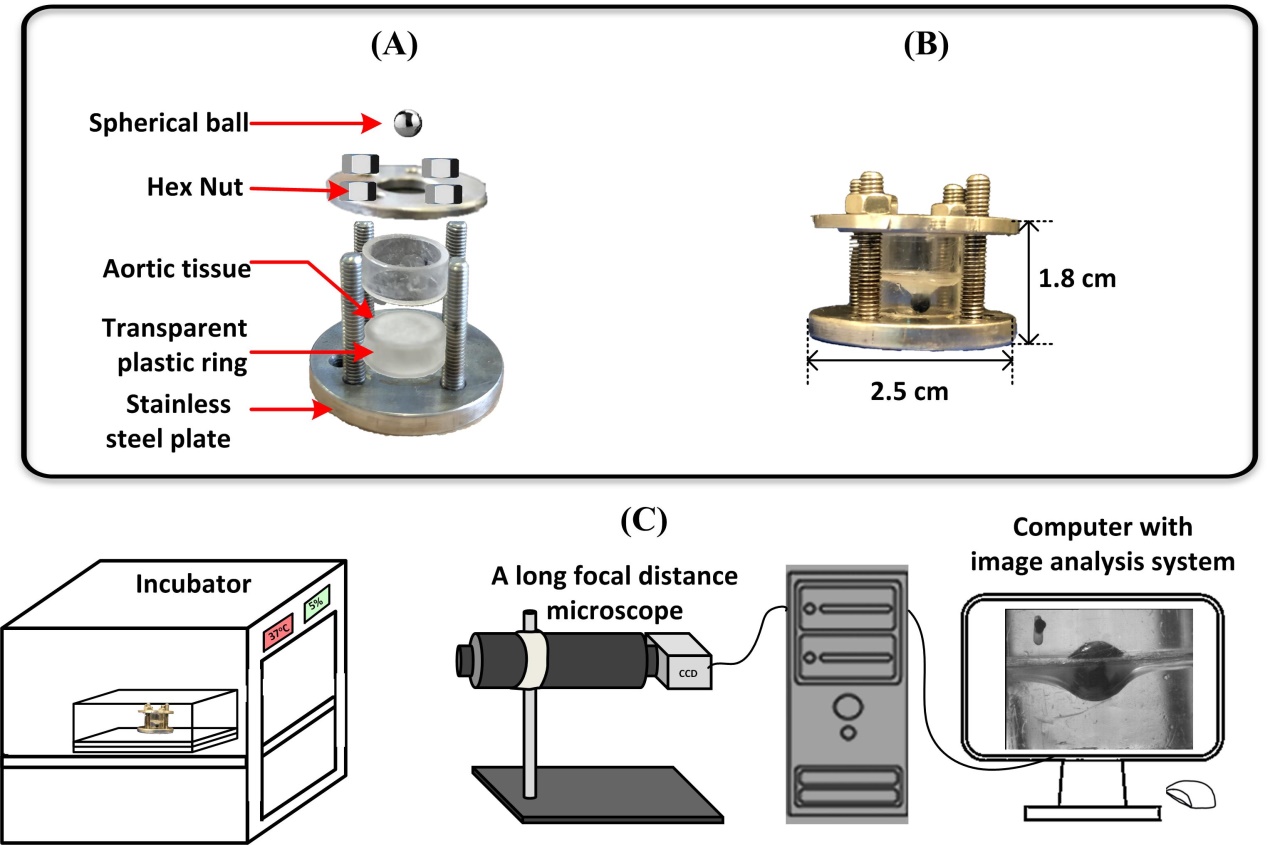


Figure S1 Schematic representation of the experimental approach for creep behaviour testing: (A) The dissembled specimen holder and spherical ball, (B) Completely assembled specimen holder and (C) Instrument system consisted of incubator; the long focal distance microscope connected with a CCD video camera and image analysis system.

Figure S2: Correlation between elastic modulus and central deformation with interval of index event to operation (IIEO) for FP (n=10 samples), TL (n=5 samples) and FL (n=4 samples) are shown A-C and D-F, respectively (Spearman product-moment correlation coefficients: R_s_).

Figure S3: Correlation between biochemical data with IIEO for FP, TL and FL: (A-C) collagen, (D-F) GAG, (G-I) elastin and (J-L) collagen/elastin ratio.

Table S1: Statistical comparison between tissue aetiology (Marfan syndrome or non-syndromic) and location (ascending or descending) within groups for biomechanical data (elastic modulus and central deformation) for FP, TL and FL. Data are displayed as median (IQR) values.

| **Tissue type** | **Central deformation (μm)** | | **P-value** | **Elastic Modulus (kPa)** | | **P-value** |
| --- | --- | --- | --- | --- | --- | --- |
|  |  | |  |  | |  |
|  | **Tissue aetiology** | |  | **Tissue aetiology** | |  |
|  | Marfan syndrome | Non- syndromic syndromic |  | Marfan syndrome | Non- syndromic |  |
| **FP** | 0.61(0.15) | 0.52(0.07) | NS | 53.5(19.5) | 63.1(13.9) | NS |
|  |  |  |  |  |  |  |
| **TL** | 0.75 | 0.74(0.04) | NS | 50.7 | 48.4(7.9) | NS |
|  |  |  |  |  |  |  |
| **FL** | 0,9(0.1) | 0,9(0.1) | NS | 42.8(1.6) | 38.1(1.8) | NS |
|  |  |  |  |  |  |  |
|  |  |  |  |  |  |  |
|  | **Location** | |  | **Location** | |  |
|  | Ascending | Descending |  | Ascending | Descending |  |
| **FP** | 0.5 | 0.6(0.1) | NS | 63.1 | 73.5(14.8) | NS |
|  |  |  |  |  |  |  |
| **TL** | 0.7 | 0.7(0.01) | NS | 51.8 | 47.9(7.6) | NS |
|  |  |  |  |  |  |  |
| **FL** | 0.9(0.01) | 0.9(0.04) | NS | 41.3(4.6) | 39.6(4.8) | NS |
|  |  |  |  |  |  |  |
